# Impella use in real-world cardiogenic shock patients: Sobering outcomes

**DOI:** 10.1371/journal.pone.0247667

**Published:** 2021-02-26

**Authors:** Khaled Q. A. Abdullah, Jana V. Roedler, Juergen vom Dahl, Istvan Szendey, Hendrik Haake, Lars Eckardt, Albert Topf, Bernhard Ohnewein, Peter Jirak, Lukas J. Motloch, Bernhard Wernly, Robert Larbig

**Affiliations:** 1 Division of Cardiology, Hospital Maria Hilf Mönchengladbach, Mönchengladbach, Germany; 2 Department of Cardiology, RWTH Aachen University, Aachen, Germany; 3 Division of Electrophysiology, Department of Cardiovascular Medicine, University of Münster, Münster, Germany; 4 Clinic II for Internal Medicine, University Hospital Salzburg, Paracelsus Medical University, Salzburg, Austria; 5 Department of Anaesthesiology, Perioperative Medicine and Intensive Care Medicine, Paracelsus Medical University, Salzburg, Austria; 6 Center for Public Health and Healthcare Research, Paracelsus Medical University, Salzburg, Austria; IRCCS Policlinico S.Donato, ITALY

## Abstract

**Background:**

Critically ill patients with cardiogenic shock could benefit from ventricular assist device support using the Impella microaxial blood pump. However, recent studies suggested Impella not to improve outcomes. We, therefore, evaluated outcomes and predictors in a real-world scenario.

**Methods:**

In this retrospective single-center trial, 125 patients suffering from cardiac arrest/cardiogenic shock between 2008 and 2018 were analyzed. 93 Patients had a prior successful cardiopulmonary resuscitation. The primary endpoint was hospital mortality. Associations of covariates with the primary endpoint were assessed by univariable and multivariable logistic regression. Adjusted odds ratios (aOR) and optimal cut-offs (using Youden index) were obtained.

**Results:**

Hospital mortality was high (81%). Baseline lactate was 4.7mmol/L [IQR = 7.1mmol/L]. In multivariable logistic regression, only age (aOR 1.13 95%CI 1.06–1.20; p<0.001) and lactate (aOR 1.23 95%CI 1.004–1.516; p = 0.046) were associated with hospital mortality, and the respective optimal cut-offs were >3.3mmol/L and age >66 years.

Patients were retrospectively stratified into three risk groups: Patients aged ≤66 years and lactate ≤3.3mmol (low-risk; n = 22); patients aged >66 years or lactate >3.3mmol/L (medium-risk; n = 52); and patients both aged >66 years and lactate >3.3mmol/L (high-risk, n = 51). Risk of death increased from 41% in the low-risk group, to 79% in the medium risk group and 100% in the high-risk group. The predictive abilities of this model were high (AUC 0.84; 95% 0.77–0.92).

**Conclusion:**

Mortality was high in this real-world collective of severely ill cardiogenic shock patients. Better patient selection is warranted to avoid unethical use of Impella. Age and lactate might help to improve patient selection.

## 1.0 Introduction

### 1.1 Background

In critically ill patients with cardiogenic shock (CS), ventricular assist devices protect against systemic hypotension and tissue hypofunction and thus could help to improve outcomes. The Impella transvalvular microaxial blood pump (Abiomed, Danvers, USA) is a commonly used assist device in this population for active LV unloading, sometimes combined with VA-ECMO. Impella supports hemodynamic stabilization and provides intermediate support by increasing cardiac output up to 3–5.0 L/min depending on the generation of the assist device. All Impella pumps, except the Impella 5.0 can be inserted percutaneously into the femoral artery and create a non-pulsatile, forward flow into the aorta. Previous studies have shown that Impella improves MAP [1], reduces LV-Load, end-diastolic pressure, oxygen consumption and myocardial work [2]. The ISAR-SHOCK Trial showed that Impella provided numerous hemodynamic improvements including cardiac index, cardiac output, MAP 30 minutes after implantation with reversal of serum lactate values [3].

Importantly, some previous studies have shown improved survival in patients with medical therapy refractory CS [4,5]. However, the mortality in patients with cardiogenic shock with a need for an assist device is high, and the therapy with Impella also bears the potential for sometimes severe complications such as reduced platelet aggregation, mechanical hemolysis, acquired von Willebrand syndrome, high purge pressures, sensor failure, suction episodes, device thrombosis, and aortic or mitral valve injury [1,6]. Other access-related complications include bleeding and vascular complications such as limb ischemia, pseudoaneurysm, and arteriovenous fistula [7]. A very recent, propensity-matched and registry-based, retrospective study by Dhruva et al. showed that the use of a microaxial LVAD in CS due to myocardial infarction had a higher risk of in-hospital death [8]. Other data from a collective of 204 patients with CS showed a strong correlation of lactate and outcome, however, they did not include patients with prior resuscitation [9]. In conclusion, it remains unclear which parameters might be helpful in real-world collectives that include patients with CS and prior successful resuscitation.

We, therefore, analyzed patients with CA/CS treated with Impella at our hospital to describe outcomes and predictors of mortality in a contemporary real-world collective.

## 2.0 Methods

### 2.1 Study design and setting

In this retrospective single-center trial, we analyzed 125 consecutive patients (36 female, 69.0±18.0 years) with CA/CS shock that were admitted to our intensive care unit (ICU) from 8/2007-7/2018. Ninety-three patients had prior successful CPR.

### 2.2 Eligibility

We included patients with cardiac arrest and/or CA/CS as determined by a systolic blood pressure below 90 mmHg for more than 30 minutes, the presence of elevated serum lactate values >2mmol/L or continuous hemodynamic instability despite inotrope or vasopressor therapy that required the implantation of an Impella blood pump. Impella position was routinely checked every 12 h in our ICU using transthoracic echocardiography by a skilled cardiologist and optimized when necessary. CA/CS in our collective was due to myocardial infarction or endstage heart failure in dilatative cardiomyopathy. The source of the medical records, samples, data regarding medication, results from diagnostic tests as well as the history of concomitant diseases was the patient database of Kliniken Maria Hilf GmbH, Mönchengladbach, Germany. The data was stored and organized using the MetaVision Software (iMDsoft, Israel). The primary endpoint of this study was hospital mortality.

### 2.3 Variables

We assessed the associations of outcome with age, lactate concentrations and CPR duration before Impella implantation.

### 2.4 Statistical analysis

The statistical analysis was carried out blindly by our statistical analytic team using the SPSS 22 software (IBM Corp, Armonk, NY, USA). Descriptive statistics were obtained for study variables. All categorical variables were compared by using the Fisher exact test. Continuous data are presented as median and interquartile range [IQR] values and variables were compared using the Mann-Whitney U test. Logistic regression was used to evaluate associations with the primary endpoint. Odds ratios (OR) and adjusted odds ratios (aOR) with 95% confidence intervals (CI) were obtained. For a multivariable logistic regression model, confounders with a p-value <0.10 in the univariate analysis were included, then a backward variable elimination was performed.

The discrimination accuracy was evaluated using ROC analysis and the c-index (area-under-the-curve (AUC)) as a cumulative measure. An optimal cut-off was calculated by means of the Youden index. We performed a sensitivity analysis in the patients with previous successful cardiopulmonary reanimation, and with acute myocardial infarction (AMI) as primary underlying pathology for the CS.

All tests were two-sided and P-values <0.05 were considered statistically significant.

### 2.5 Ethics

The local ethical board of the Aerztekammer Nordrhein Westfalen approved this study (Approval Nr.: 154/2019). The study conformed with the principals outlined in the declaration of Helsinki. All data were fully anonymized befor evaluation. Our Ethics committee waived the requirements for informed consent. The authors received no specific funding for this work.

## 3.0 Results

### 3.1 Baseline characteristics in survivors vs. non-survivors

The majority of our patients were male (71.2%, n = 89) and had risk factors for coronary artery disease (CAD, 87.2%, n = 109) such as arterial hypertension (HTN, 60.8%, n = 76) and diabetes mellitus (DM, 25.6%, n = 32). Patients with HFrEF (41.6%, n = 52) and structural heart disease (20.0%, n = 25) were frequent. Median left ventricular ejection fraction was 25% [25%] as assessed by echocardiography using eyeballing and the biplane Simpson method. No significant differences in the distribution of comorbidities or the underlying pathologies of CA/CS between survivors and non-survivors were found (Table 1). 81 (65%) of our patients received a coronary angiography and stenting. Survivors of cardiogenic shock were significantly younger (53.50 [17.3] years vs. 71.0 [16.0] years; p<0.001), and baseline lactate of all patients was 4.7 [7.1mmol/L]. In survivors, we found lower baseline lactate of 2.1mmol/L [4.6mmol/L] as compared to non-survivors with 5.4mmol/L [6.7mmol/L] (Table 1). CPR duration was not associated with the outcome in our collective (Table 1). The type of Impella device (Impella 2.5, n = 91; Impella CP, n = 31; Impella 3.5, n = 3) was also not associated with mortality which was high (Impella 2.5, 84%; Impella CP, 79%; Impella 3.5, 100%; p = 0.59) in all devices.

**Table 1:** Baseline characteristics and laboratory parameters before assist device implantation.

	Total population n = 125		In-hospital survival n = 24		In-hospital mortality n = 101		p-value
	*n*	*Median (Q3-Q1) or %*	*n*	*Median (Q3-Q1) or %*	*n*	*Median (Q3-Q1) or %*	
Gender (female)	36	28.8%	6	25.0%	30	29.7%	0.803
Age	125	69.0 (18.0)	24	53.50 (17.25)	101	71.0 0(16.00)	<0.001
LVEF (%)	101	25.0 (25.0)	22	32.5 (32.5)	79	20.0 (20.0)	0.131
Survived CPR	93	74.4%	12	50.0%	81	79.3%	0.004
Duration of CPR (minutes)	90	20.00 (22.75)	24	25.00 (36.00)	78	20.00 (22.75)	0.761
Cardiogenic shock due to MI	100	80.0%	18	75.0%	82	81.2%	0.571
**Medical History**							
Arterial hypertension	76	60.8%	14	58.3%	62	61.4%	0.819
Diabetes mellitus	32	25.6%	3	12.5%	29	28.7%	0.124
CAD	109	87.2%	21	87.5%	88	87.1%	>0.999
PAD	9	7.2%	0	0.0%	9	8.9%	0.205
HFrEF	52	41.6%	8	33.3%	44	43.6%	0.365
HFpEF	5	4.0%	0	0.0%	5	5.0%	0.582
Valvular heart disease	14	11.2%	1	4.2%	13	12.9%	0.302
Structural heart disease	25	20.0%	4	16.7%	21	20.8%	0.782
Pulmonary hypertension	8	6.4%	3	12.5%	5	5.0%	0.180
COPD	11	8.8%	1	4.2%	10	9.9%	0.689
Malignancy	16	12.8%	2	8.3%	4	4.0%	0.735
**Laboratory**							
Creatinine (mg/dl)	125	1.50 (0.80)	24	1.30 (0.80)	101	1.60 (0.70)	0.157
Haemoglobin (g/dl)	125	12.80 (3.40)	24	12.55 (3,54)	101	12.90 (3.95)	0.508
Creatine kinase (U/l)	122	332.00 (979.00)	23	240.00 (577.00)	99	366.0 0(1063.00)	0.060
GOT (U/l)	123	131.00 (230.00)	24	65.50 (66.00)	99	155.00 (237.00)	0.006
GPT (U/l)	123	70.00 (124.00)	24	55.00 (62.75)	99	80.00 (138.00)	0.136
Lactate (U/l)	125	4.70 (7.10)	24	2.10 (4.63)	101	5.40 (6.65)	0.001

Baseline characteristics of the study population: CAD = coronary artery disease, COPD = chronic obstructive pulmonary disease, CPR = cardiopulmonary resuscitation, HFpEF = heart failure with preserved ejection fraction, HFrEF = heart failure with reduced ejection fraction, LVEF = left ventricular ejection fraction, MI = myocardial infarction, PAD = periphery artery disease, PH = pulmonary hypertension. The raw data is accessible in the supporting information (S1 Table).

The overall hospital mortality was high, with 81%. In the sub-group analysis of patients with previous CPR and acute myocardial infarction (AMI) as underlying pathology, the mortality rates were 87% and 82%. None of the concomitant diseases was associated with this endpoint. In univariable logistic regression previous CPR (OR 4.05 95%CI 1.59–10.35; p<0.003), age (OR 1.11 95%CI 1.06–1.16; p<0.001) and lactate (OR 1.26 95%CI 1.08–1.48; p = 0.004) were the only parameters associated with hospital mortality (Table 2). However, after performing the multivariable logistic regression analyses, only age (aOR 1.13 95%CI 1.06–1.20; p<0.001) and lactate (aOR 1.23 95%CI 1.004–1.516; p = 0.046) remained as indicators associated with hospital fatality events (Table 3).

**Table 2:** Univariate regression analyses.

	OR	95% CI	p-value
*Age (per year)*	1.11	1.06–1.16	<0.001
*LVEF (per %)*			>0.100
*Previous CPR (yes/no)*	4.05	1.59–10.35	0.003
Cardiogenic shock due to MI *(yes/no)*			>0.100
*Arterial hypertension (yes/no)*			>0.100
Diabetes mellitus *(yes/no)*			>0.100
*CAD (yes/no)*			>0.100
*PAD (yes/no)*			>0.100
*HFrEF (yes/no)*			>0.100
*HFpEF (yes/no)*			>0.100
*Valvular heart disease (yes/no)*			>0.100
*Structural heart disease (yes/no)*			>0.100
*PH (yes/no)*			>0.100
*COPD (yes/no)*			>0.100
*Malignancy (yes/no)*			>0.100
*CK (per U/L)*			>0.100
*Creatinine (per mg/dL)*	1.93	0.88–4.23	0.100
*Hb (per g/dL)*			>0.10
*GOT (per U/L)*	1.02	1.00–1.01	0.090
*GPT (per U/L)*			>0.10
*Lactate (per mmol/L)*	1.26	1.08–1.48	0.004

Results of the univariate regression analyses: CAD = coronary artery disease, CI = confidence interval, COPD = chronic obstructive pulmonary disease, CPR = cardiopulmonary resuscitation, HFpEF = heart failure with preserved ejection fraction, HFrEF = heart failure with reduced ejection fraction, LVEF = left ventricular ejection fraction, MI = myocardial infarction, PAD = periphery artery disease, OR = odds ratio, PH = pulmonary hypertension. The raw data is accessible in the supporting information (S1 Table).

**Table 3:** Multivariate regression analyses.

	aOR	95% CI	p-value
*Age (per year)*	1.13	1.06–1.20	<0.001
*Previous CPR (yes/no)*	2.25	0.59–8.50	0.230
*Creatinine (per mg/dL)*	1.01	0.44–2.33	0.980
*GOT (per U/L)*	1.003	1.000–1.006	0.053
*Lactate (per mmol/L)*	1.23	1.004–1.516	0.046

Results of the multivariate regression analyses: aOR = adjusted odds ratio, CI = confidence interval, CPR = cardiopulmonary. The raw data is accessible in the supporting information (S1 Table).

**Fig 1:**
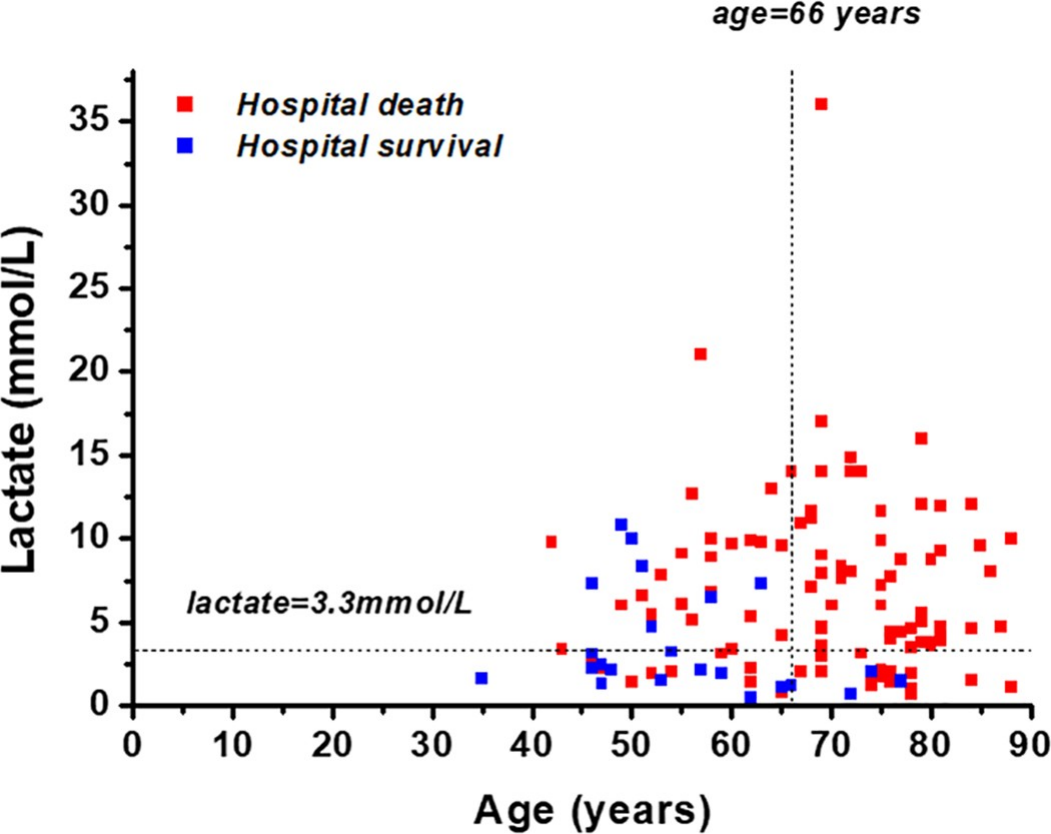
Scatter plot of age, lactate and survival. Scatter plot of both in hospital survivors (blue) and deceased patients (red) in relation to age and lactate levels. The raw data is accessible in the supporting information (S1 Table).

Both age (AUC 0.80 95%CI 0.72–0.86) and lactate (AUC 0.73 95%CI 0.64–0.80) had high discrimination accuracy, the optimal cut-offs were >66 years and >3.3 mmol/L, respectively. Patients above 66 years (n = 72; 94% vs. 62%; p<0.001; Fig 1) and patients with an initial lactate concentration >3.3mmol/L (n = 82; 92% vs. 61%; p<0.001; Fig 1) evidenced significantly higher hospital mortality.

**Fig 2:**
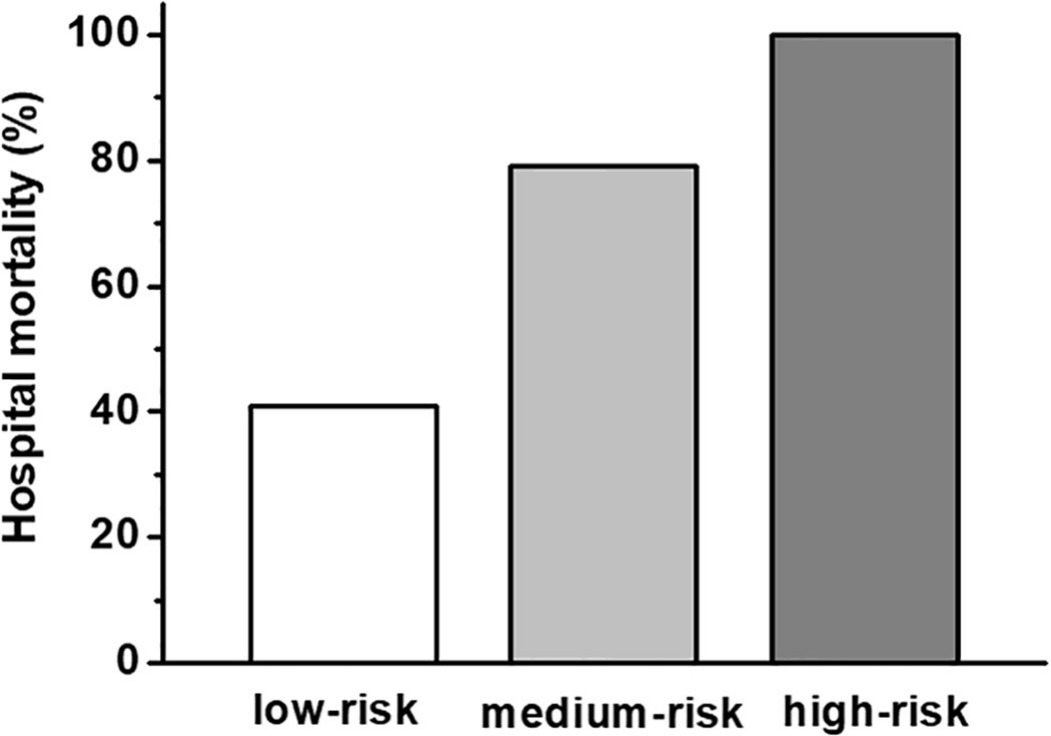
Hospital survival rates in stratified risk groups. Patients aged ≤66 years and lactate ≤3.3mmol (low-risk; n = 22); patients aged >66 years or lactate >3.3mmol/L (medium-risk; n = 52); and patients both aged >66 years or lactate >3.3mmol/L (high-risk, n = 51). The raw data is accessible in the supporting information (S1 Table).

**Fig 3:**
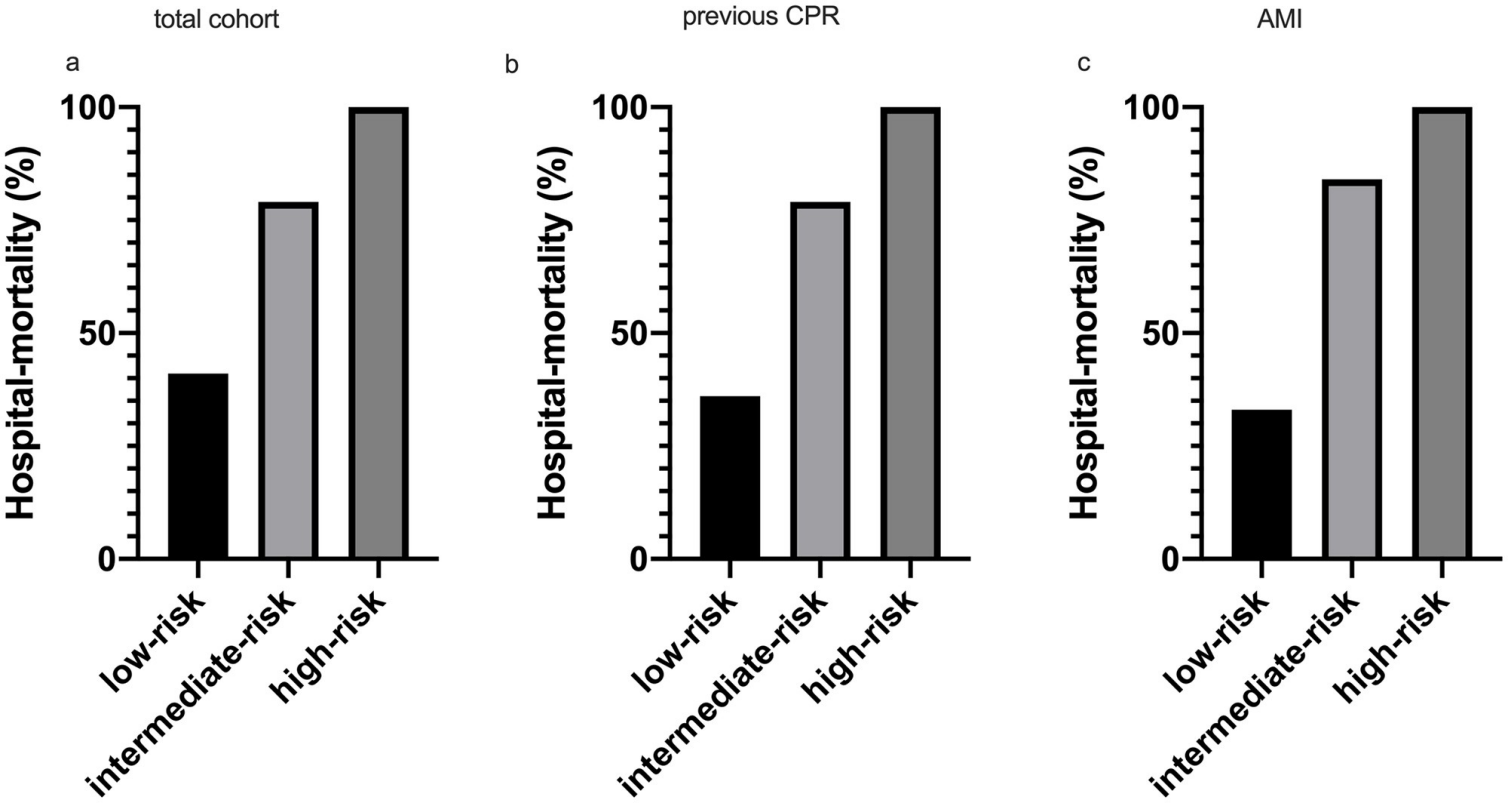
Survival in different sub-groups. Hospital survival rates in different sub-groups of CA/CS: a) the total cohort, b) patients with previous successful cardiopulmonary resuscitation (previous CPR) and c) acute myocardial infarction (AMI). The raw data is accessible in the supporting information (S1 Table).

The patients were retrospectively stratified into three risk groups: Patients aged ≤66 years and lactate ≤3.3mmol (low-risk; n = 22); patients aged >66 years or lactate >3.3mmol/L (medium-risk; n = 52); and patients both aged >66 years and lactate >3.3mmol/L (high-risk, n = 51). The risk of death increased from 41% in the low-risk group to 79% in the medium-risk group and 100% in the high-risk group (Figs 2 and 3a; p<0.001). The predictive abilities of this model were high (AUC 0.84 95% 0.77–0.92).

The sensitivity analysis in patients with previous CPR revealed a high predictive ability (AUC 0.88 95%CI 0.79–0.96). In patients with previous CPR, the risk of death increased from 36% in the low-risk group to 79% in the medium-risk group and 100% in the high-risk group (Fig 3b; p<0.001). Also, in the patients with an AMI as primary underlying etiology, the model performed well (AUC 0.85 95%CI 0.77–0.93). In the patients with an AMI as primary underlying etiology, the risk of death increased from 33% in the low-risk group to 84% in the medium-risk group and 100% in the high-risk group (Fig 3c; p<0.001).

## 4.0 Discussion

Our study sought to analyze the outcomes, and the impact of baseline characteristics in real-world patients with CA/CS treated with an Impella assist device. The hospital mortality was high, while lactate and age were shown to be the only variables associated with hospital mortality. Indeed, other baseline variables, including concomitant diseases, were not associated with this endpoint. The death rate was 100% in the high-risk group across all sub-groups.

This is in accordance with previous trials. Age had a strong impact on the outcome in our collective, which was also found in previous studies [10]. Hyperlactatemia is commonly used in intensive care medicine as a marker for end-organ function [11,12]. Previous studies have demonstrated its predictive value in the prediction of patient mortality in the setting of septic shock [11]. Furthermore, a strong correlation of serum lactate and CS in the Impella collective of Rohm et al. was revealed [9]. However, the authors did not assess the impact of a previous successful CPR, which is a frequent etiology of cardiogenic shock in this population. Therefore, in our study, we included both successful CPR survivors and CS of other origins. We found a strong association between baseline lactate values as an indicator of end-organ function and hospital survival in our collective using multivariable analysis. In our analysis in sub-groups with previous CPR and acute myocardial infarction we were able to confirm these results. Based upon this we considered it feasible to combine patients with CA and CS for pragmatic and statistical reasons despite differing underlying pathologies. Other investigators presented similar results [9,12–15]. Indeed, survivors in our study also had a lower baseline-lactate value as suggested in the trials above or the Euroshock II study [10]. However, in our collective, 74.4% (93) of patients had a previous CPR. Of note, this percentage of patients was higher as compared to the Euroshock II trial, which also observed a significant influence of this parameter on fatal outcomes, again underlining the predictive value of lactate in severely ill CA/CS patients [10].

Additionally, only the Impella 2.5 pump was used in the Euroshock II trial, while in our cohort also the Impella 3.5 and CP were applied. We found no significant impact of Impella type on survival. This underlines the limitation of strategies restricted to provide hemodynamic stability through a high performing blood pump in CS.

Overall, in comparison to previous trials reporting a mortality rate of 64.2% in CS [10], in our real-world scenario, we observed a high mortality of 81%. However, as already discussed, previous CPR was frequent in our population.

Therefore, our findings might suggest that in a severely ill, real world collective of CA/CS the beneficial impact of an invasive assist device therapy might be even more limited as previously suggested. This underlines the importance of patient selection to provide the best medical care for specified populations. Indeed, Impella assist device therapy is limited due to the invasive nature of this procedure. Therefore, ethical consideration should be warranted when proceeding with this strategy in a high-risk population with a limited chance of survival. In our trial, by applying cut-off values for lactate and age, we were able to identify risk groups. Of note, this suggestive strategy could help to guide intensive care physician in daily clinical practice. We propose the application of an Impella guided strategy in younger patients (aged ≤66) and lower baseline lactate levels (lactate ≤3.3mmol/L) while this approach should be reconsidered when in older CS/CA patients high baseline lactate is revealed (aged >66 years and lactate >3.3mmol/L).

### 4.1 Conclusion

The mortality rate was high in this real-world collective of severely sick cardiogenic shock patients. Better patient selection is warranted to avoid unethical use of Impella. Age and lactate might help to guide decision making in clinical practice. Nevertheless, due to the heterogeneity of our real-world collective, these results must, however, be individually scrutinized in each patient and should not lead to an inadequate holdback of Impella therapy in selected patients.

### 4.2 Limitations

This study has several limitations due to its single-center and retrospective design with a small patient number. Additionally, we included patients with CA/CS that in 93 cases have had a CPR. The combination of these collectives could lead to an overestimated mortality due to the poor outcome in patients with CPR. However, this combination is common in a real-world setting, additionally we found similar results in our sub-group analysis with regard to selected pathologies.

## Supporting information

S1 Table (XLSX)
